# An Accurate Image Measurement Method Based on a Laser-Based Virtual Scale

**DOI:** 10.3390/s19183955

**Published:** 2019-09-13

**Authors:** Shiping Huang, Wei Ding, Yonghui Huang

**Affiliations:** 1 School of Civil Engineering and Transportation, South China University of Technology, Guangzhou 510640, China; ctasihuang@scut.edu.cn (S.H.); ctwding@mail.scut.edu.cn (W.D.); 2 State Key Laboratory of Subtropical Building Science, South China University of Technology, Guangzhou 510640, China; 3 Guangzhou University – Tamkang University Joint Research Center for Engineering Structure Disaster Prevention and Control, Guangzhou University, Guangzhou 510006, China

**Keywords:** non-contacting measurement, full-field scale, laser virtual scale, moving least squares

## Abstract

Image measurement methods have been widely used in broad areas due to their accuracy and efficiency. However, current techniques usually involve complex calibration, an elaborate optical design, or sensitivity to the test environment. In this paper, a simple optical device was designed to emit parallel beams to obtain a virtual scale for measurement purposes. The proposed theory ensures the robustness of the system when obtaining each scale in the presence of uncertainty. The scale creates a mapping from image coordinates to world coordinates. By using the moving least squares (MLS) method, a full-field scale map can be reconstructed to achieve high-precision measurement at the sub-pixel level. Experimental verifications are carried out, showing that the proposed method provides very accurate and reliable results. The proposed approach is simple in terms of equipment, and the scale can be automatically calculated. Therefore, the system proposed in this paper is a promising candidate as a tool for non-contacting measurements (e.g., the crack development, geometric size) in the inaccessible structures such as high-rise buildings and long-span bridges.

## 1. Introduction

Modern industry requires higher accuracy and efficiency for geometric measurement instruments [1]. Traditional geometric measurement instruments (e.g., ruler, calipers) as a contacting measurement technique cannot measure complex geometry or fragile objects, or inaccessible places. Therefore, in the last few decades, optical technology as a non-contacting measurement technique has been wildly used in broad areas. In general, the optical measurement techniques fall into two categories [2]: the methods that use laser beams, such as laser doppler vibrometry [3], electronic speckle pattern interferometry (ESPI) [4], and digital speckle shearography (DSS) [5]; and the methods that use white light, which is also called photogrammetry or image measurement method.

Due to the rapid development of the microcomputer, memory chip, and camera sensors, image measurement method became an effective and practical tool for geometric measurement, which has been commonly accepted and widely used in the fields of experimental mechanics and civil engineering. Based on the type of targets, they can be categorized into three groups [2]: point tracking method [6,7], digital image correlation (DIC) method [8], and target-less method [9]. The point tracking technique uses digital cameras to distinguish the coordinates of discrete points mounted to the targets. Based on the position of the discrete points, it can measure the geometric information of the targets. The point tracking method is usually used to monitor the displacement of the target point. Instead of a set of discrete points’ measurements, the DIC method is to provide the full-field geometric information with sub-pixel accuracy [10,11]. The accuracy of the DIC method is closely related to the quality of the speckle pattern, environmental vibration, and camera calibration [12]. Therefore, these factors limit the application of the DIC method, such as crack development in bridges and high-rise buildings, where on-site environmental vibration continually occurs and some structures are inaccessible. As for the target-less method, researchers utilize the internal features or edges of a structure to recognize the object or areas of the object that needs to be tracked. This method is especially useful when it is impossible to mount optical targets to the structure or spray the speckle pattern on the surface. 

Even though non-contacting methods have achieved great success in the last few decades, there are still some challenges in practical applications. Harsh on-site environments limit the application of current methods. For example, camera vibration due to a vehicle passing or natural excitation in bridges makes it impossible for DIC method to complete the test. The optical target or calibration target cannot be fixed to the inaccessible structures such as high-rise buildings and long-span bridges. Furthermore, the accuracy of the current measurement techniques usually relies on the intrinsic and extrinsic parameters of the system, which requires a strict calibration process [13,14].

To this end, we propose a simple laser setup for non-contacting two-dimensional measurements. The laser emits parallel beams to form spot pairs (recorded by the camera), which is subsequently used to reconstruct a full-field scale map. The scale map bridges the image coordinates to a world coordinate system, making sub-pixel accuracy possible. In the following sections, we will first set up the equipment, and then the MLS method [15,16,17] will be introduced to reconstruct a full-field scale map. Finally, we present two experiments to verify the proposed method.

## 2. Design of the Device

### 2.1. Device Setup

**Figure 1:**
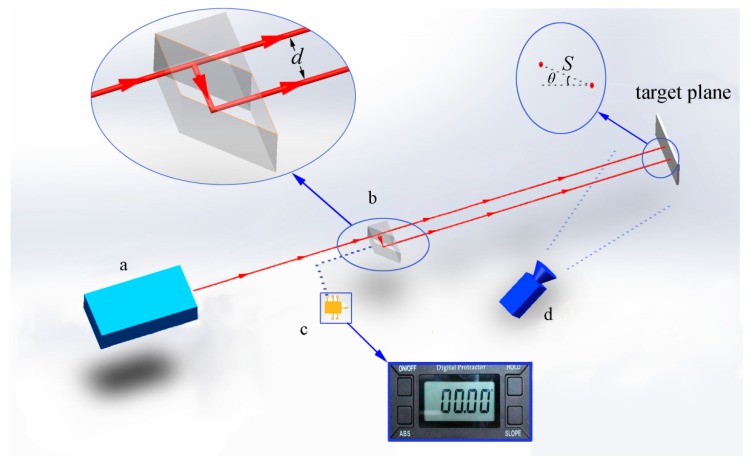
Laser virtual scale system: (**a**) semiconductor laser; (**b**) lateral displacement beam splitter; (**c**) inclinometer; and (**d**) camera.

To obtain the mapping from image coordinates to a world coordinate system, the following device has been developed. The device consists of four modules: (1) a semiconductor laser emitter; (2) a beam splitter; (3) an inclinometer; and (4) a digital camera, as shown in Figure 1. These four modules work independently and have been encapsulated separately. The semiconductor laser emitter is used to emit a laser beam with a circular shape. The semiconductor laser emitter in the current design should meet the following requirement: (1) the output power is sufficient (generally between 0.5 to 5 mW), thus it can be recorded by the camera; (2) small beam divergence (generally less than 1 mrad), which indicates the beam size will not be too big in the target plane; and (3) circular shape, which is used to obtain α in the next section. The lateral displacement beam splitter is to divide the laser beam into two parallel beams with a fixed distance, which is further used as the virtual scale for measurement purposes. The inclinometer is used to measure the angle of the spot pair’s direction in the next section, and we suggest the accuracy is better than ± (0.1° + 1%). The camera module is to record the image with laser scale. The camera is fixed. Each laser scale is recorded by a single image. Since the camera is fixed, the images have the same coordinate systems. Combining all the laser scales in different locations builds the full-field scale image. Obviously, the camera quality (high CCD sensitivity, high resolution, and good lens) will affect the measuring accuracy. However, in this device, we only use the in-expensive camera. The detailed device work process is as follows.

A semiconductor laser emits a beam (circular shape) first, and then the beam goes through a lateral displacement beam splitter. The lateral displacement beam splitter outputs two identical parallel beams separated by a fixed distance (this distance is called beam separation and denoted as d). The beam splitter consists of a precision rhomboid prism cemented to a right angle prism. The current manufacturing technology can easily ensure that the exiting beams are parallel within 30 arcsec, which indicates that the distance of the parallel beams (d) varies very little along the path to the target plane.

The two beams are finally projected on the target plane and formed two spots (a spot pair), which is recorded by the fixed camera. After the distance between the spots (spot pair distance, denoted as S) is obtained, it is used to establish the relation between the image coordinates and the world coordinate system. It is noted that image coordinates represent coordinates in terms of pixel values in the image plane.

Since the scale (ratio of the image coordinates to the world coordinates) is nonlinear across the image field, we need a series of scales to construct a full-field scale map. The scales can be achieved by moving the laser, rotating the beam splitter at different locations. These scales are recorded by a fixed camera. The advantages of this device are as follows: the system does not need the camera parameters to obtain the relationship from the image coordinate to the world coordinate system, and thus it does not need camera calibration.

In the current design, the laser emits visible light (the wavelength is between 400–760 nm). However, the device is not limited to visible light. Invisible light and harmful laser light can also be used when the infrared camera is used. As mentioned before, the more parallel the beams separated by the splitter are, the more accurate the virtual scale will be. Therefore, more accurate beam splitters will be used when the manufacturing technology develops. Further hardware improvement of the system will be done in our future work.

### 2.2. Spot Pair Distance (S) Calculation Theory

**Figure 2:**
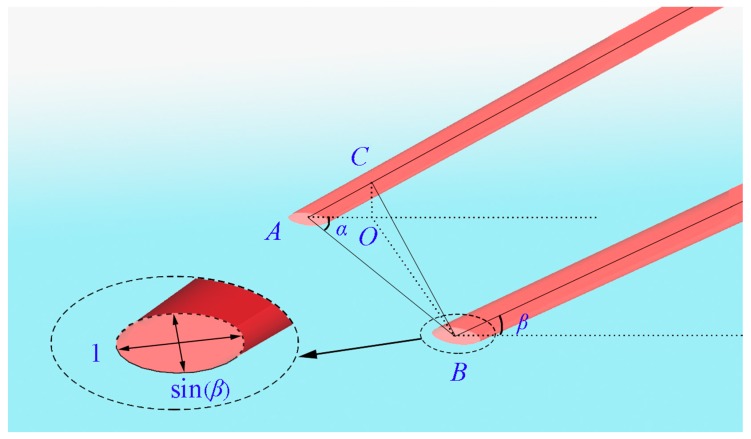
Calculation of *S* for the device in the target plane.

If the device above generates parallel beams perpendicular (*β* = *π*/2, as seen in Figure 2) to the image plane, the beam separation (denoted as *d, d* = *BC*) is the spot pair distance (denoted as *S, S* = *AB*) in the target plane, i.e., *d* = *S*. Note that distance or physical distance is different from pixel distance. However, in many cases, the output angle *β* is not a right angle and depends on the on-site environment, and thus, *S* should be calculated using the following theory. In this section, we use basic optical principles to obtain the spot pair distance (*S*). Let us assume that the two beams intersect with the image plane at points *A* and *B* (with angle *β*), as shown in Figure 2. When the beams are perpendicular (*β* is equal to *π*/2) to the target plane, the spots are circular. When *β* is not equal to *π*/2, the circle becomes an ellipse. Obviously, the angle *β* can be obtained via the ratio *r* of the spot’s semiminor axis and the semimajor axis via the expression sin(*β*) = *r*, as shown in Figure 2. Note that the distance between the beams is the length of the line *BC*. The projection of point *C* on the image plane is point *O*. Since we set the angle *BAO* to be *α*, which can be adjusted by rotating the beam splitter and measured in the image coordinates, then we have the following relationship according to the cosine theorem

(1) $$BO^{2}=AO^{2}+AB^{2}-2AB\times AO\times \cos(\alpha )$$

Obviously, the angles *AOC* and *BOC* are right angles. Thus, we have

(2) $$\begin{array}{l} BC^{2}=BO^{2}+AO^{2}\tan^{2}(\beta )\\ AC^{2}=AO^{2}+AO^{2}\tan^{2}(\beta ) \end{array}$$

The angle between lines *BC* and *AC* is a right angle. Therefore, we have

(3) $$AB^{2}=AC^{2}+BC^{2}=BO^{2}+2AO^{2}\tan^{2}(\beta )+AO^{2}$$

Substituting *BO* in Equation (1) into Equation (3), we obtain

(4) $$AO=\frac{AB\cos(\alpha )}{1+\tan^{2}(\beta )}$$

Finally, we have the following expression for *S*

(5) $$S=AB=BC/\sqrt{1-\cos^{2}(\alpha )\cos^{2}(\beta )}$$

**Figure 3:**
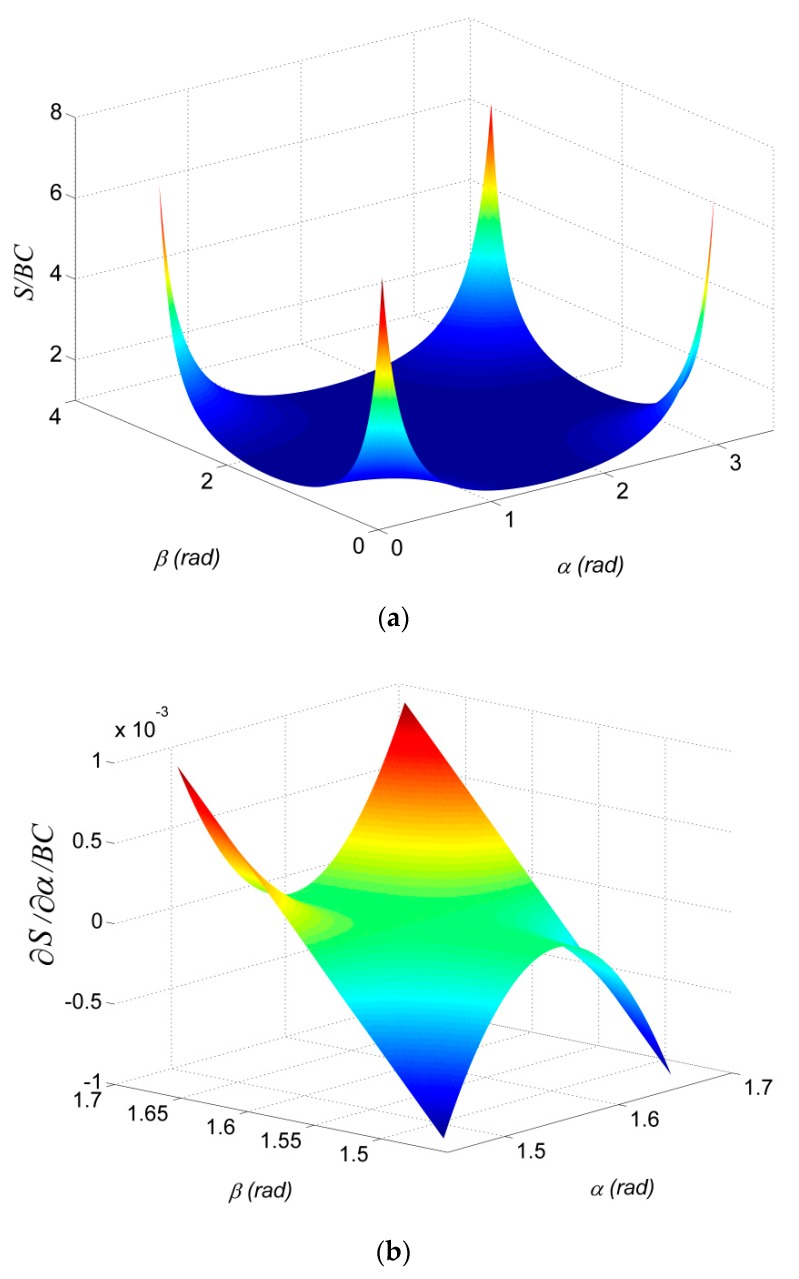
Sensitivity analysis of *β* and *α* to *S*: (**a**) *S* distribution with varying *β* and *α*; (**b**) Sensitivity of *S* due to perturbations of *β* and *α* at π/2.

According to Equation (5), when cos^2^(*α*) = 0 or cos^2^(*β*) = 0, the length of the line *AB* is equal to *BC*, i.e., *S* = *d*. In practical applications, the target object may be unapproachable, and *β* may not be equal to *π*/2. In this case, we can adjust *α* to be *π*/2 via rotating the beam splitter to make *S* = *d*. As observed in Equation (5), different values of *α* and *β* lead to different *S*. As we demonstrated in Figure 3a, where *S* is normalized by *BC*, changing the angles *α* and *β* from *π*/2 to 0 (or *π*/2 to *π*) results in the length of *S* increasing from 1 to infinity. Although *S* can be obtained by controlling either *α* or *β* to be *π*/2, the small perturbations of *α* or *β* cannot be avoided in practical applications. The sensitivity of parameter *α* or *β* to *S* can be obtained by taking the partial derivative of Equation (5) with respect to *α* or *β* as

(6) $$\frac{\partial S}{\partial \alpha }=-\frac{BC\cdot \cos(\alpha )\sin(\alpha )\cos^{2}(\beta )}{\sqrt{{\left(1-\cos^{2}(\alpha )\cos^{2}(\beta )\right)}^{3}}}$$

It is noted that ∂S/∂β it has the same result as the Equation (6) after exchange *α* to *β*. Figure 3b demonstrated the result for ∂S/∂α or ∂S/∂β when *α* or *β* close to *π*/2, i.e., *α* or *β* at the interval [*π*/2 − 0.1, *π*/2 + 0.1], which shows that the perturbations of *α* or *β* have a small effect on *S* (the maximum perturbation for *S* is less than one thousandth). This calculation theory ensures the robustness of the system in the presence of uncertainty. 

## 3. Numerical Method of the Full-Field Scale Map

### 3.1. Theory

**Figure 4:**
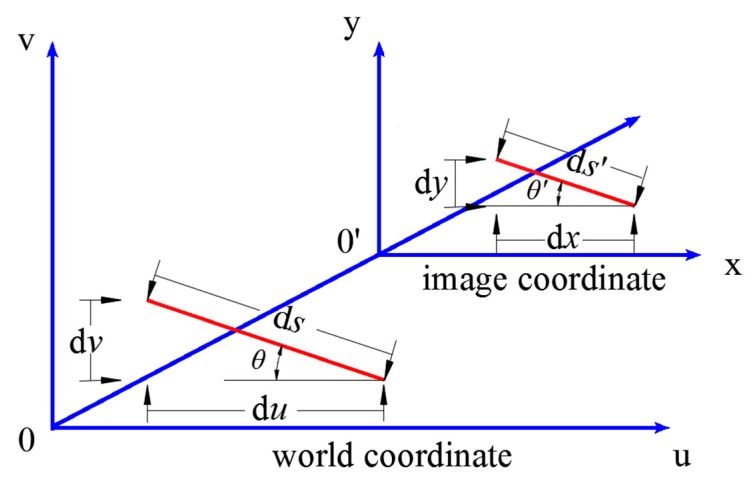
Mapping from world coordinate (space plane) to image coordinate (image plane).

To obtain the real size of the object, we need to construct a mapping from the image coordinates to the world coordinate system (as seen in Figure 4). Here, for simplicity, the image coordinate origin, which is conventionally located in the upper-left corner, was shifted to the center (parallel to the world coordinate), as seen in Figure 4. Let us assume that we have a small segment d*s* with an incline angle *θ*; thus, its projection into the horizontal segment d*u* and vertical segment d*v* can be expressed as

(7) $$\begin{array}{l} du=ds\cdot \cos\theta \\ dv=ds\cdot \sin\theta \end{array}$$

where *θ* is measured by the inclinometer cemented to the beam splitter. 

The segment d*s* has been recorded in the image, where its pixel length is denoted as d*s’*. Accordingly, the horizontal pixel segment d*x* and vertical pixel segment d*y* can be expressed as

(8) $$\begin{array}{l} dx=ds^{\prime }\cdot \cos\theta^{\prime }\\ dy=ds^{\prime }\cdot \sin\theta^{\prime } \end{array}$$

where $\theta^{\prime }=\arctan(\frac{y_{i1}-y_{i0}}{x_{i1}-x_{i0}})$, and (*x_i_*_1_, *y_i_*_1_) and (*x_i_*_0_, *y_i_*_0_) are the coordinates of the spots’ centroids in the image coordinate system.

Therefore, the relation between the image coordinates and the world coordinate system can be expressed as

(9) $$\begin{array}{l} du=dx\cdot h(x,y)\\ dv=dy\cdot g(x,y) \end{array}$$

where (*x*, *y*) is the middle point of d*s’*, *h*(*x*, *y*) is the measured scale in the *x* direction, and *g*(*x*, *y*) is the measured scale in the *y* direction.

In the device, the measured scale *h*(*x*, *y*) at (*x_i_*, *y_i_*), which is located at the middle point of the line formed by the two centroids of the laser spots, represents the scale for d*s’* in an average sense. According to Equation (9), the scales at this point can be expressed as

(10) $$h(x_{i},y_{i})=\frac{S_{x}}{\left|dx\right|}=\frac{S_{x}}{\left|x_{i1}-x_{i0}\right|}$$

(11) $$g(x_{i},y_{i})=\frac{S_{y}}{\left|dy\right|}=\frac{S_{y}}{\left|y_{i1}-y_{i0}\right|}$$

where $x_{i}=\frac{x_{i1}+x_{i0}}{2}$, $y_{i}=\frac{y_{i1}+y_{i0}}{2}$; $S_{x}$ and $S_{y}$ are the horizontal and vertical projections of the spot pair distance (*S*).

Through the set *h*(*x_i_*, *y_i_*)and *g*(*x_i_*, *y_i_*), we can reconstruct the full-field scale map. With the scale map, we can measure all the geometry inside the image. Many methods are used to reconstruct the surface [18]. Among these methods, piecewise low-order fitting and polynomial fitting are the two most frequently used tools. However, high-order polynomial fitting can lead to ill-conditioned matrices, while piecewise low-order fitting results in discontinuities. To overcome the difficulty mentioned above, we introduce the moving least squares (MLS) method [19]. MLS method is a method of reconstructing continuous functions from a set of unorganized point samples via the calculation of a weighted least squares measure biased towards the region around the point at which the reconstructed value is requested. The MLS method is useful for reconstructing a surface from a set of points. In the following section, we use measured scale *h*(*x_i_*, *y_i_*) to reconstruct the scale map *H*(*x*, *y*) in the x direction. Suppose the scale function (i.e., MLS approximant) *H*(*x*, *y*) consists of polynomial and undetermined coefficients as

(12) $$H(x,y)=\sum_{i=1}^{m}p_{i}(x,y)a_{i}(x,y)=p^{T}(x,y)a(x,y)$$

where *p_i_*(*x*, *y*) is a complete monomial basis of order *m* and *a_i_*(*x*, *y*) is the undetermined coefficient. Since the undetermined coefficients are location-dependent, Equation (12) can be rewritten at the subdomain centered at (*x*, *y*) as $H((\bar{x},\bar{y}),(x,y))$

(13) $$H((\bar{x},\bar{y}),(x,y))=\sum_{i=1}^{m}p_{i}(\bar{x},\bar{y})a_{i}(x,y)=p^{T}(\bar{x},\bar{y})a(x,y)$$

where $\left(\bar{x},\bar{y}\right)$ are the points in the subdomain centered at (*x*, *y*). Since the scale is nonlinear, we use a quadratic basis as

(14) $$p^{T}=[1,x,y,x^{2},xy,y^{2}]$$

In this case, *m* is equal to 6. The weight function can be expressed as

(15) $$w_{I}(x,y)=w((x,y)-(x_{I},y_{I}))$$

where (*x_I_*, *y_I_*) is the known node, ((*x*, *y*)- (*x_I_*, *y_I_*)) represents the distance between the undetermined point and the node, i.e., $\sqrt{{\left(x-x_{I}\right)}^{2}+{\left(y-y_{I}\right)}^{2}}$.

**Figure 5:**
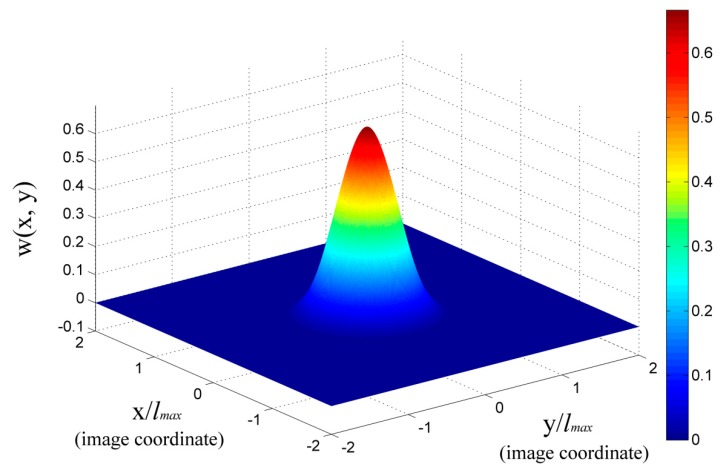
Cubic spline weight function distribution.

There are a number of weight functions that are used for the MLS method. Here, we use a cubic spline function as

(16) $$w_{I}(\bar{l})=\left\{ \begin{array}{ll} \frac{2}{3}-4{\bar{l}}^{2}+4{\bar{l}}^{3} & \left(\bar{l}\leq \frac{1}{2}\right)\\ \frac{4}{3}-4{\bar{l}}^{1}+4{\bar{l}}^{2}-\frac{4}{3}{\bar{l}}^{3} & \left(\frac{1}{2}<\bar{l}\leq 1\right)\\ 0 & \left(\bar{l}>1\right) \end{array}\right.$$

where $\bar{l}=l/l_{\max}$ is the normalized distance, *l* is the distance and $l_{\max}$ is the influential radius. The characteristic of the cubic spline weight function is demonstrated in Figure 5, where we set the unit along *X* and *Y* axis equal to $l_{\max}$, and (*x_I_*, *y_I_*) equal to (0, 0).

**Figure 6:**
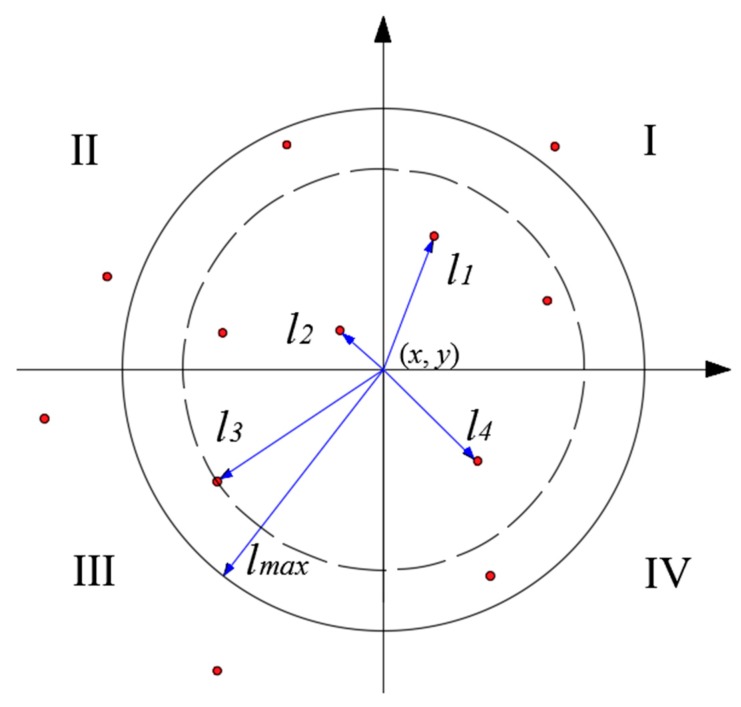
Quadrant method for the influential radius.

To avoid the singularity in the MLS algorithm, $l_{\max}$ is set to be a proper value to include sufficient nodes in the subdomain [20]. In this paper, the influential radius is determined by the quadrant method [21]. $l_{\max}$ is obtained via $l_{\max}=k\cdot \max(l_{1},l_{2},l_{3},l_{4})$, where *k* is the positive number between 1.2 to 2.5 and *l_i_* is the nearest distance from the interpolation point (*x*, *y*) to the known node for each quadrant, respectively, as seen in Figure 6.

Note that the local approximant $H((\bar{x},\bar{y}),(x,y))$ can be expressed by the known nodes (i.e., $\left(\bar{x},\bar{y}\right)$ is replaced by $\left(x_{I},y_{I}\right)$). The weighted sum of squared errors at all nodes has the following form

(17) $$\begin{array}{ll} J & =\sum_{I=1}^{N}w_{I}(x,y){\left[H((x_{I},y_{I}),(x,y))-h(x_{I},y_{I})\right]}^{2}\\ & =\sum_{I=1}^{N}w_{I}(x,y){\left[p^{T}(x_{I},y_{I})a(x,y)-h(x_{I},y_{I})\right]}^{2} \end{array}$$

where *N* is the node number, and it is required $w_{I}(x,y)>0$.

To obtain the best approximant of $H((\bar{x},\bar{y}),(x,y))$, *J* can be minimized with respect to ***a***(*x*, *y*) as

(18) $$\frac{\partial J}{\partial a(x,y)}=2\sum_{I=1}^{N}w_{I}(x,y)[p^{T}(x_{I},y_{I})a(x,y)-h(x_{I},y_{I})]p^{T}(x_{I},y_{I})=0$$

Thus,

(19) $$\sum_{I=1}^{N}w_{I}(x,y)p(x_{I},y_{I})p^{T}(x_{I},y_{I})a(x,y)=\sum_{I=1}^{N}w_{I}(x,y)p(x_{I},y_{I})h(x_{I},y_{I})$$

Equation (19) has the matrix form

(20) A(x,y)a(x,y)=B(x,y)h

The undetermined coefficient a(x,y) can be expressed as

(21) $$a(x,y)=A^{-1}(x,y)B(x,y)h$$

where

(22) $$A(x,y)=\sum_{I=1}^{N}w_{I}(x,y)p(x_{I},y_{I})p^{T}(x_{I},y_{I})$$

(23) $$B(x,y)=[w_{1}(x,y)p(x_{1},y_{1}),w_{2}(x,y)p(x_{2},y_{2}),\cdots \cdots ,w_{N}(x,y)p(x_{N},y_{N})]$$

(24) $$h={\left[h(x_{1},y_{1}),h(x_{2},y_{2}),\ldots \ldots ,h(x_{N},y_{N})\right]}^{T}$$

Thus, the approximant based on MLS can be expressed as

(25) $$H(x,y)=p^{T}(x,y)A^{-1}(x,y)B(x,y)h$$

The inverse of matrix ***A*** is a critical step in the MLS algorithm. In the algorithm, we first obtain the determinant of matrix ***A***. If it is zero, the matrix ***A*** is singular and we need to increase the influential radius to include more nodes to form matrix ***A***. Alternatively, one can determine the invertibility of matrix ***A*** by checking its rank via singular value decomposition or rank-revealing QR decomposition. A full rank square matrix is invertible. There are many methods for matrix inversion and we used Gaussian elimination method in the following examples [22]. The computational efficiency of the MLS method will be discussed in our future work.

It is noted that *G*(*x, y*) (the scale map in the y direction) can be obtained in the same manner. Once the scale functions *H*(*x, y*) and *G*(*x, y*) have been obtained, the object length in the image can be calculated via the integration method with d*s*. d*s* can be expressed as

(26) $$ds=\sqrt{(H(x,y)\cdot dx{)}^{2}+{\left(G(x,y)\cdot dy\right)}^{2}}$$

Similarly, the area of the object in the image can be calculated via integration with d*A*. d*A* is expressed as

(27) dA=H(x,y)⋅dx⋅G(x,y)⋅dy

### 3.2. Algorithm

**Figure 7:**
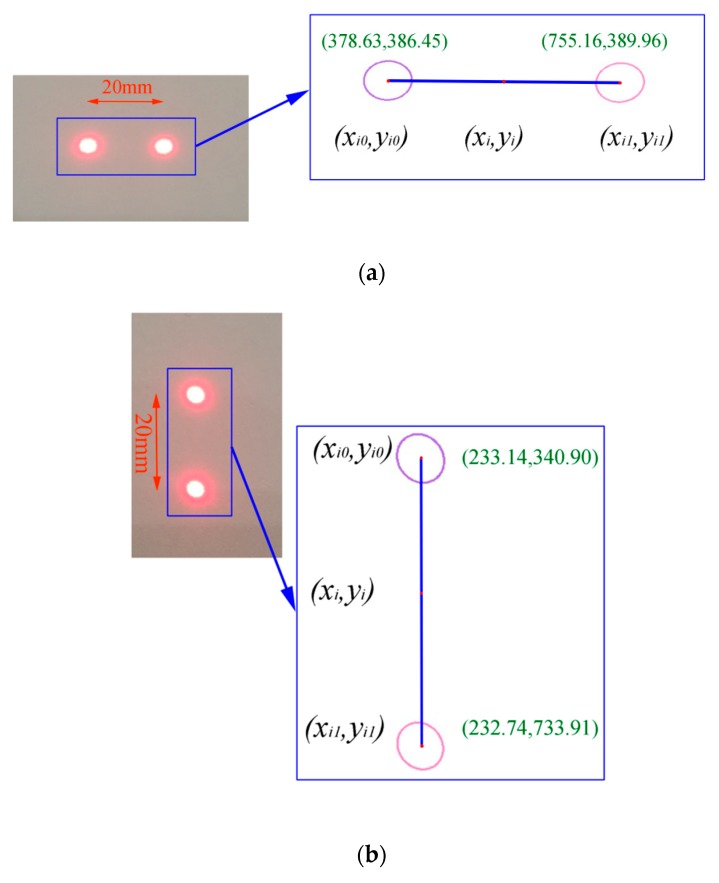
Example of the laser scales: (**a**) The horizontal spot pair, *θ* = *θ’* = 0; (**b**) The vertical spot pair, *θ* = *θ’* = π/2.

Since the spots have identical shapes, they can be recognized by the algorithm. An edge detection method [23,24] was used to detect the spot edge, as shown in Figure 7. Once the edges of the two spots have been detected, the pixel length between two spots’ centroids can be calculated. The centroids (*x_i_*_0_, *y_i_*_0_) and (*x_i_*_1_, *y_i_*_1_) are obtained via a set of discrete points (denoted as array (*x*, *y*)) on the edge as

(28) $$x_{i0}=\frac{\sum_{x,y}\left(array(x,y)\cdot x\right)}{\sum_{x,y}array(x,y)}$$

(29) $$y_{i0}=\frac{\sum_{x,y}\left(array(x,y)\cdot y\right)}{\sum_{x,y}array(x,y)}$$

Subsequently, *h*(*x_i_*, *y_i_*) and *g*(*x_i_*, *y_i_*) can be calculated via Equations (10) and (11), respectively. Finally, *H*(*x*, *y*) and *G*(*x*, *y*) can be obtained via the MLS approach.

**Figure 8:**
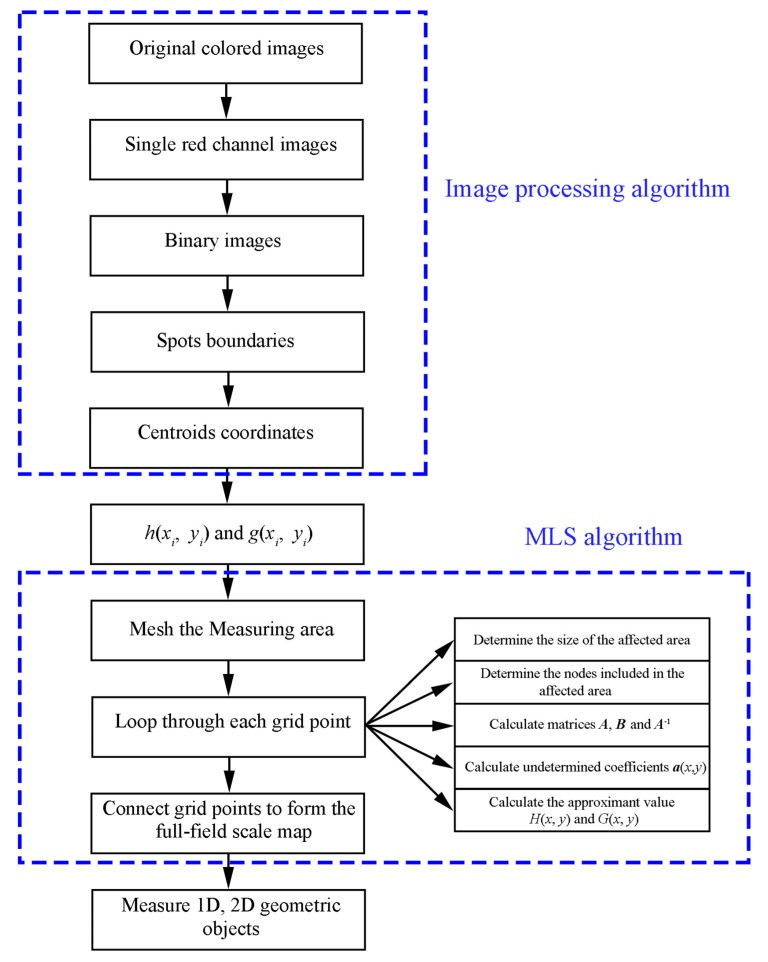
Flowchart of the proposed method.

To summarize, there are two critical steps in the proposed method. The first one is the image processing algorithm, which is used to obtain a single virtual scale. The second one is the MLS algorithm, which is used to build the full-field scale map. The detailed flowchart can be seen in Figure 8. Due to the characteristics of the device (outputting two identical spots), every single virtual scale can be calculated automatically. When the full-field scale is reconstructed by the MLS algorithm, high-precision measurement is possible. Verification will be done in the next section. 

## 4. Experimental Verification

To verify the accuracy and stability of the proposed method, we designed the following experiments. 

### 4.1. Experimental Verification of the Full-Field Sale Map

#### 4.1.1. Design of the Experiment

**Figure 9:**
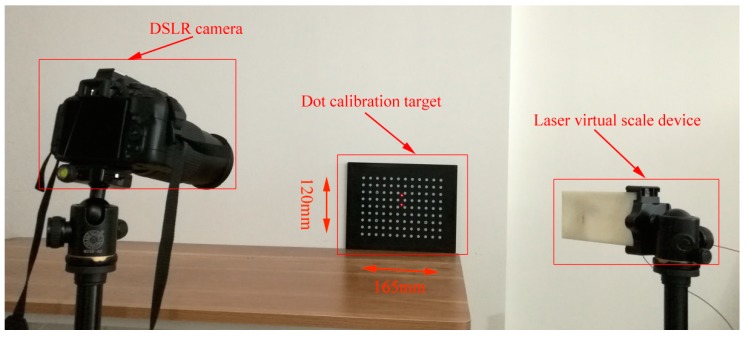
Experimental setup.

In this example, we used a dot calibration target (with 108 lattices) with known dimensions as the target. The calibration target’s specifications can be found in Table 1. The experimental setup can be seen in Figure 9. The detailed specifications of the semiconductor laser emitter, lateral displacement beam splitter and inclinometer can be found in Table 2, Table 3 and Table 4. The proposed method is used to reconstruct the full-field scale map, which is then used to compare with the scale map obtained by the calibration target. It is noted that different cameras have different intrinsic parameters, which is essential for measurement accuracy. Here, we used two different cameras, a Nikon digital single lens reflex (DSLR) camera and a Huawei cell phone camera. The detailed specifications of the cameras can be found in Table 5.

#### 4.1.2. Experimental Procedures

**Figure 10:**
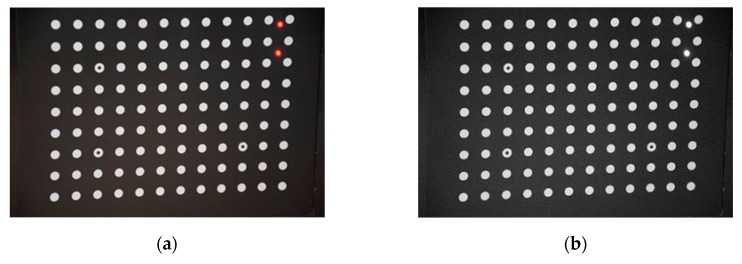

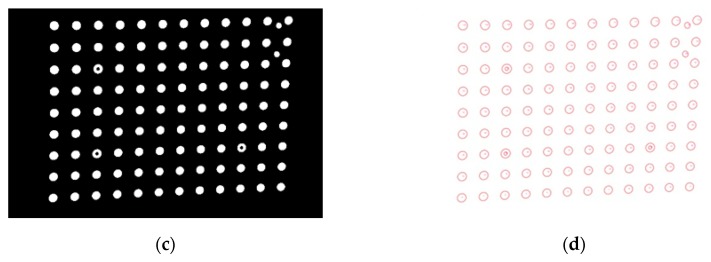
Procedures for image processing: (**a**) original colored image; (**b**) red channel image; (**c**) binary image; (**d**) boundaries of circular targets.

We used the following steps to complete the experiment:

1. Laser spots are projected to the target plane (calibration target), which is recorded by the camera. For simplicity, we projected the vertical laser spot pair (*α* = *π*/2, *θ* = *π*/2) for comparison.
2. Repeat step 1 by moving the laser spots until enough laser spot pairs have been generated. In this case, we obtained 7 × 11 = 77 laser spot pairs.
3. Use the algorithm to get the coordinates of each laser spot pair to obtain laser scale at different locations, as shown in Figure 10.
4. Use the MLS method to reconstruct the scale map based on the discrete laser scale points.

Meanwhile, each dot’s coordinate of the calibration target is known, and the scale map can be easily obtained as follows, which is referred to as the direct method. At each lattice, the scale is equal to the ratio of the physical distance to the pixel distance between two neighboring dots. Using the MLS interpolation, then the scale map can be reconstructed based on the scale at each lattice.

#### 4.1.3. Verification of the Full-Field Scale Map

**Figure 11:**
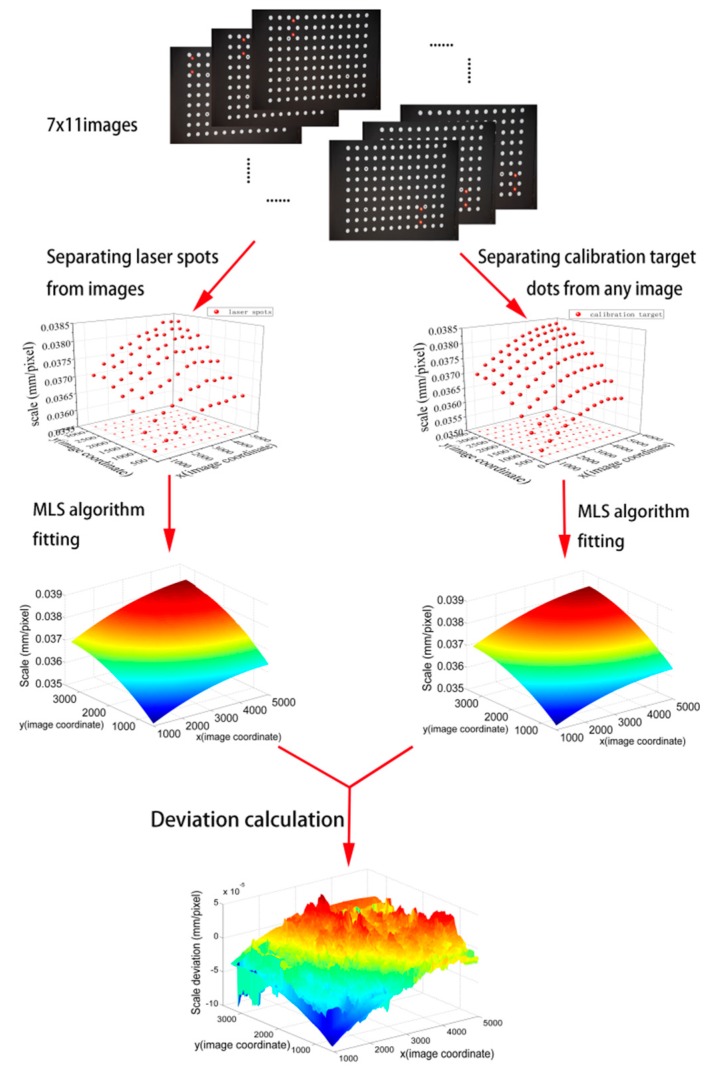
Procedures for experimental data processing.

**Figure 12:**
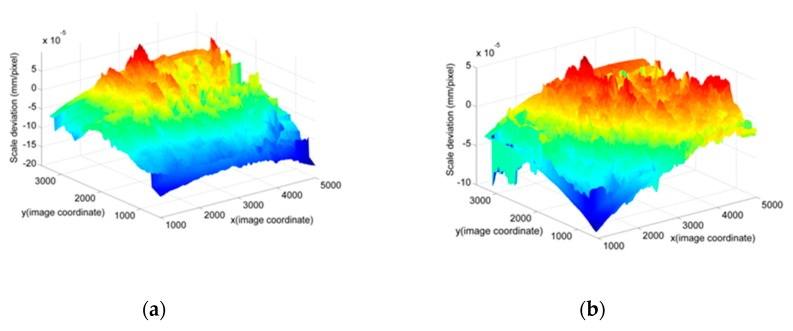

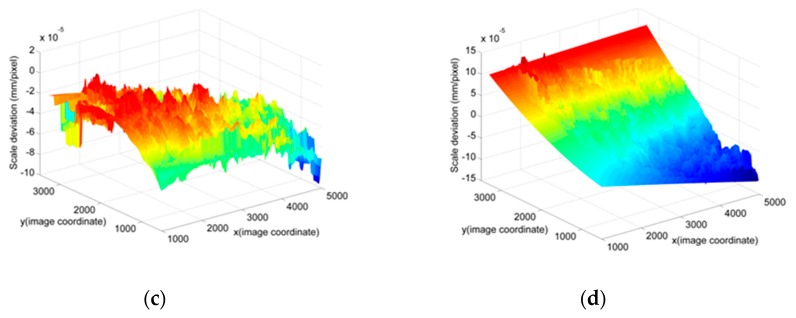
Deviations for different camera angles based on the DSLR camera: (**a**) The camera angle is 0; (**b**) Camera angle is 12.5°; (**c**) Camera angle is 25°; (**d**) Camera angle is 45°.

**Figure 13:**
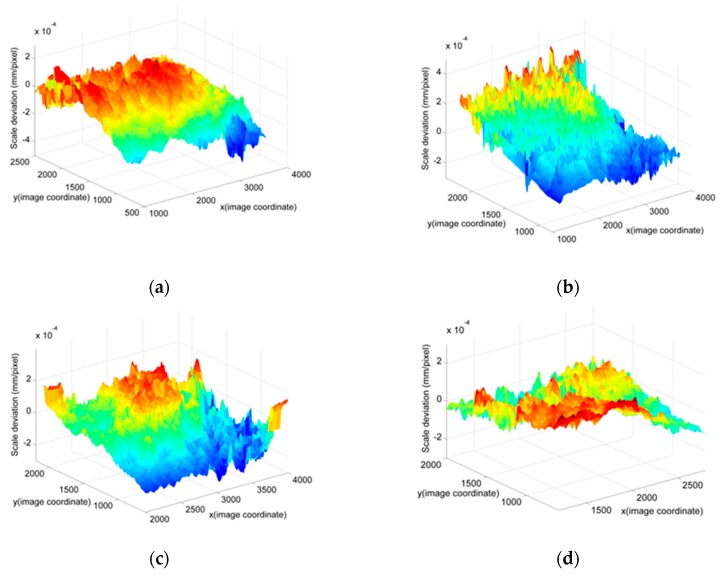
Deviations for different camera angles based on Huawei camera: (**a**) The camera angle is 0; (**b**) camera angle is 12.5°; (**c**) camera angle is 25°; (**d**) camera angle is 45°.

The full-field scale map by the proposed method is compared to the direct measurement scale map by the calibration target. The data processing flow chart can be seen in Figure 11. Deviation (the difference between the proposed method and the direct method) is used to evaluate the result. In Figure 11, the full-field deviation map for *G* (*x*, *y*) is demonstrated. The result is slightly affected by the camera angles (the angles between the camera-target direction and the calibration target’s normal direction), as seen in Figure 12. The camera angles are set to be 0, 12.5°, 25°, and 45°, respectively. The deviation is within the range of ±0.5%, as shown in Figure 12 and Table 6. There are three main reasons for this. The first one comes from the manufacturing errors of the calibration target, which is 3–5 μm. The second one comes from the distortion and nonlinearity of the image, which depends on the camera intrinsic parameters. Adding more laser spot pairs to obtain more laser scales can reduce this effect. For comparison, we changed the Nikon camera to Huawei cell phone camera. It was found that the error was increased to ±1%, as shown in Figure 13 and Table 7. This is because the cell phone camera’s quality (Huawei) is not as good as that of the Nikon DSLR camera. The third one comes from the error of the spot pair distance calculation, which will be discussed in the next section by an experiment.

#### 4.1.4. Local Accuracy of the Laser Scale 

**Figure 14:**
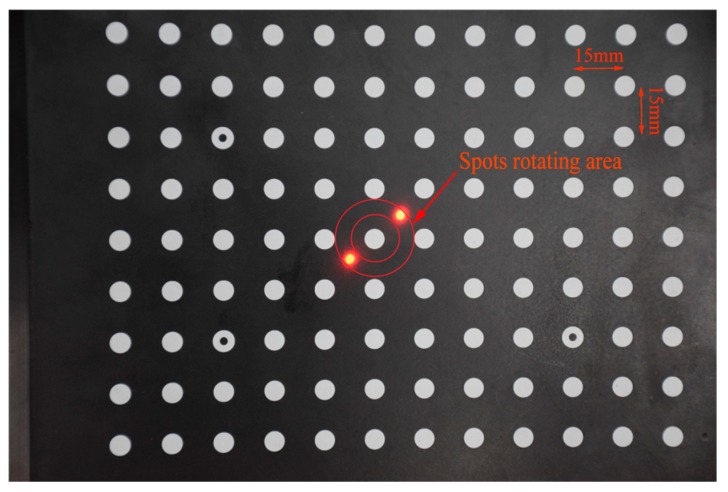
Experimental result of the spot pair.

**Figure 15:**
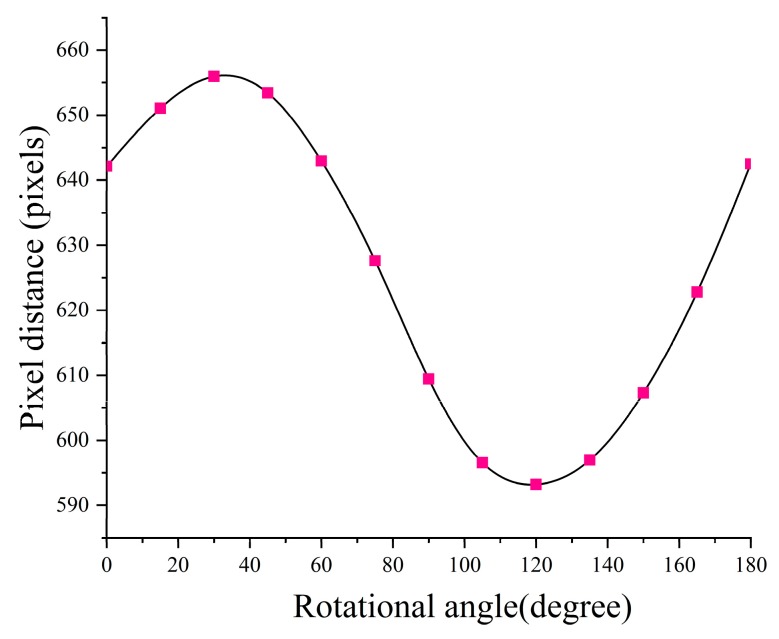
Orientation (angle *a*) effect on the spot pair distance (pixel distance).

Accuracy of the local laser scale is the foundation of the full-field scale accuracy. The spot pair distance accuracy is affected by the angle *α* and *β*, which is theoretically discussed in Section 2.2. In practical applications, we usually get the spot pair distance by controlling the angle *α*. Here, an experiment was carried out for verification. The experimental setup is as follows. Firstly, we fixed the camera and the calibration target. The target is with a distance of 2 m to the laser and the camera was fixed in place with a distance of 2.5 m to the target. The camera angle is 12.5°. By rotating the laser system for different *α*, we get a set of laser spot pairs, as shown in Figure 14. In the local area, the scale of the spot pair distance to the pixel distance is considered to be linear. We compared the pixel distance (in Figure 15) and picked up the minimum pixel distance orientation as the spot pair orientation for *α* = *π*/2. In Figure 15, it can be observed that the spot pair orientation effect on its distance agreed with Equation (5). By measuring the spot pair distance (*S*) for *α* = *π*/2, it is almost equal to *d*, with 0.015% difference. The reason for this comes from two factors. The first one is that the laser beams are not perfectly paralleled; the second one is that the pixel’s distance in the spot pair rotating area is nonlinear.

### 4.2. Measurement of the Curved Wire

**Figure 16:**
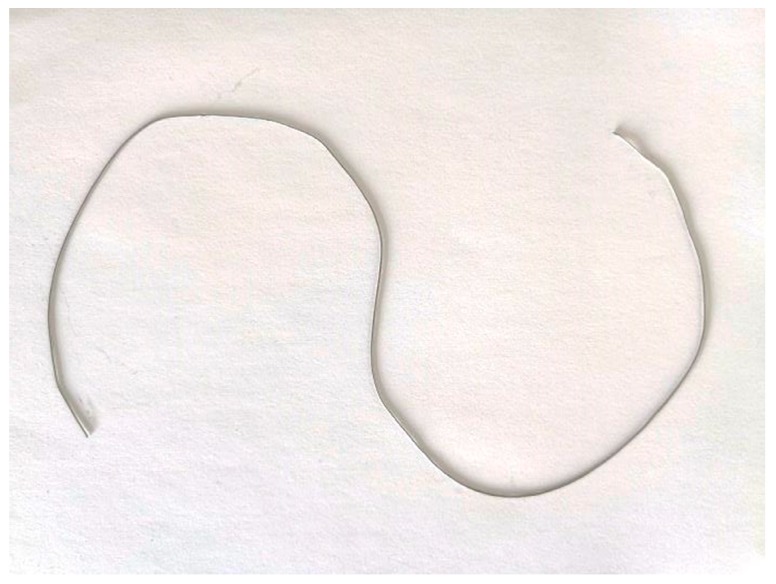
The measured wire.

**Figure 17:**
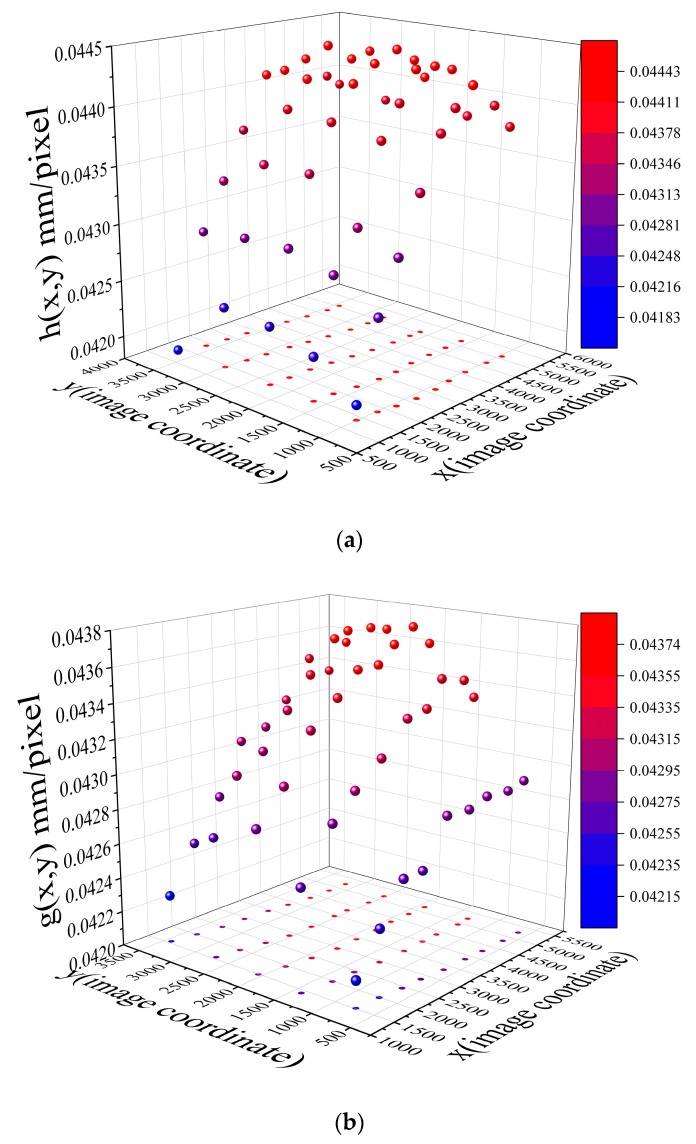
Experimental data: (**a**)*h*(*x_i_*,*y_i_*) result; (**b**) *g*(*x_i_,y_i_*) result.

This test is to measure the length of a wire, which is formed by folding a 500-mm-long tin wire, as shown in Figure 16. The wire was attached to a board with a distance of 2m to the laser. The camera was fixed in place with a distance of 2.5 m to the board. The camera angle is 0. We moved the laser, and the spots were formed at different locations on the board. The fixed camera recorded those spots. By controlling the incline angle *θ*, we recorded 45 values of *h*(*x_i_*, *y_i_*) and 45 values of *g*(*x_i_*, *y_i_*), as shown in Figure 17.

**Figure 18:**
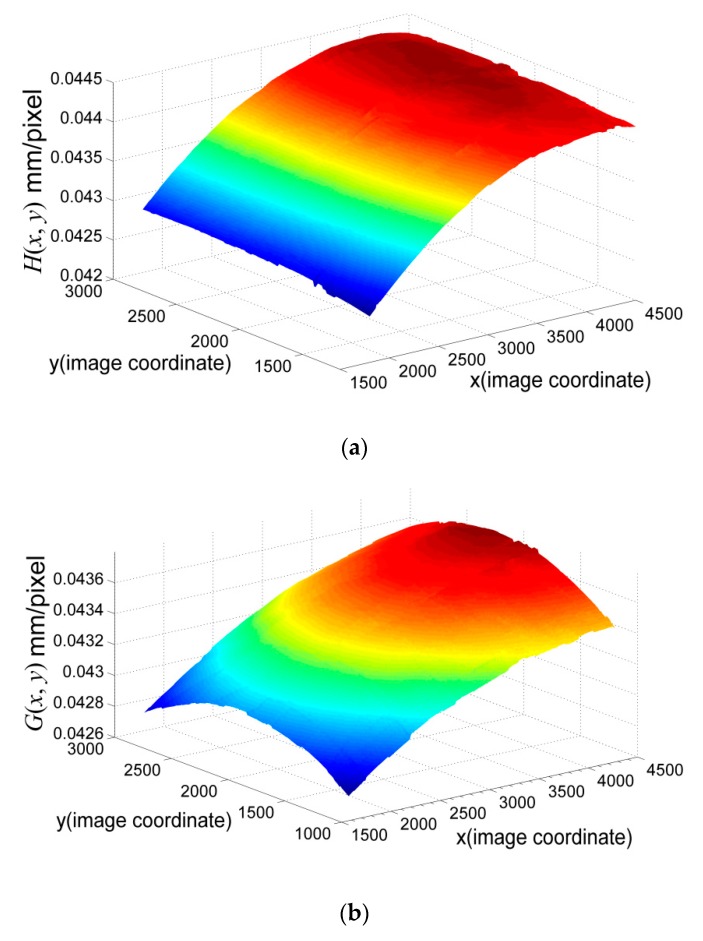
Full-field scale map using MLS method: (**a**) *H*(*x, y*)map; (**b**) *G*(*x, y*)map.

By using a set of *h*(*x_i_*, *y_i_*)and *g*(*x_i_*, *y_i_*) in MLS, the full field of the scale was obtained, as shown in Figure 18. According to Equation (26), the wire was divided into 100 elements and 200 elements, respectively, for integration. The integration results for these two cases are almost the same, which indicates that d*s’* (its corresponding horizontal pixel segment is d*x* and vertical pixel segment d*y*) is sufficiently small. Using the proposed method, the wire length was 501.02 mm, with an error tolerance less than 0.3%. When using the scale at the center of the image (uniform scale), the wire length was 504.58 mm, with an error tolerance less than 1%. Obviously, using MLS to reconstruct the full-field scale for the image can give much better results. This also indicates the presence of the nonlinearity of the scale in the image. The detailed results can be found in Table 8.

## 5. Conclusions

In this paper, we proposed a new method for non-contacting image measurements. By designing laser-based virtual scale equipment, a set of scales to reconstruct a full-field scale map for an image is obtained. The scale map bridges image coordinates to a world coordinate system and can be used to measure the real size of an object in the image. Experimental verifications are carried out, showing that the proposed method gives very accurate and reliable results.

The method has the following advantages and potentials: (1) it does not require calibration for the camera, which is usually complex and unique to each camera; (2) the scale can be calculated automatically since the two spots are theoretically identical; (3) the scale calculation theory ensures the robustness of the system when obtaining each scale in the presence of uncertainty; (4) the full-field scale map can be reconstructed via a set of scale points and thus can be used to measure anything in the image; (5) the approach can achieve high-precision measurement at the sub-pixel level; and (6) the complexity of the scale map, which is nonlinear and location-dependent, can be easily solved by the proposed method. Thus, the system proposed in this paper is a promising candidate tool for non-contacting measurements and can be used in broad areas with high-accuracy requirements.

## Figures and Tables

**Table 1:** Main technical specifications for the dot calibration target.

Specification	Value
Dimensions	186 × 141 × 6 mm
Lattice	12 × 9
Center distance	15 mm
Dot diameter	6 mm
Material expansion coefficient	8.6 × 10^−6^/°C
Accuracy	±3–5μm

**Table 2:** Main technical specifications for the semiconductor laser emitter.

Specification	Value
Wavelength	635 nm
Output Power	0.60 mW (maximum)
Beam Diameter	3.0 mm
Beam Divergence	0.35 mrad
Beam Astigmatism	<10 μm
Output Type	Free Space
Storage Temperature	−40 °C to +80 °C
Operating Temperature	−10 °C to +40 °C

**Table 3:** Main technical specifications for the lateral displacement beam splitter.

Specification	Value
P-Polarization Transmission	45.00%
S-Polarization Reflection	45.00%
Wavelength Range	430–670 nm
Dimensional Tolerance	±0.1 mm
Surface Flatness	λ/8
Angle Tolerance	±5 arcsec
Beam Separation	20.00 mm
Parallelism	<30 arcsec

**Table 4:** Main technical specifications for the inclinometer.

Specification	Value
Product Accuracy	±(0.05° + 1%)
Measuring Range	Single axis 360°, Biaxial ± 40°
Resolution	0.01°
Operating Temperature	0 °C to +50 °C
Storage Temperature	−10°C to +60 °C
Response Time	<0.4 second

**Table 5:** Main technical specifications for two cameras.

Specification	Value	
	DSLR Camera	Cell Phone Camera
Sensor Size	23.5 × 15.6 mm	Diagonal 6.521 mm (5.2 × 3.9 mm)
Sensor Type	CMOS	CMOS
Effective Pixels	24 megapixels	16 megapixels
Max Resolution	6000 × 4000	4608 × 3456
Focal Length	18–140 mm	4 mm

**Table 6:** Deviations for different camera angles based on the DSLR camera.

Camera Angle	Average Scale (mm/pixel)	Maximum Positive Deviation (10^−5^mm/pixel)	Maximum Negative Deviation (10^−5^mm/pixel)	Maximum Positive Deviation Permillage (‰)	Maximum Negative Deviation Permillage (‰)
0	0.0366	8.35	−16.82	2.28	−4.60
12.5°	0.0372	4.71	−9.55	1.27	−2.57
25°	0.0404	0.30	−9.69	0.07	−2.40
45°	0.0411	14.19	−11.87	3.45	−2.89

**Table 7:** Deviations for different camera angles based on Huawei camera.

Camera Angle	Average Scale (mm/pixel)	Maximum Positive Deviation (10^−4^mm/pixel)	Maximum Negative Deviation (10^−4^mm/pixel)	Maximum Positive Deviation Permillage (‰)	Maximum Negative Deviation Permillage (‰)
0	0.0467	1.57	−4.67	3.36	−10.00
12.5°	0.0517	3.89	−2.42	7.52	−4.68
25°	0.0550	2.59	−2.69	4.71	−4.90
45°	0.0569	1.98	−2.54	3.48	−4.46

**Table 8:** Errors of measurement results by different methods.

Measurement Methods	Length Measurement Results (mm)	Error Percentage (%)
Full-field scale measurement	501.02	0.204
Uniform scale measurement	504.58	0.916
